# Cerebro-Spinal Fever in the Royal Navy from August 1st, 1916 to July 31st, 1917

**Published:** 1918-03

**Authors:** 


***Cerebro-Spinal Fever in the Royal Navy from August 1st, 1916 to July 31st, 1917.*** By Temp. Surgeon-General H. D. HOLLECTON, R. N. Abs. from the *Lancet*, Jan. 19, 1918.

Statistics are presented for 143 cases of bacteriologically proved cerebro-spinal fever in which the meningococcus was demonstrated in the spinal fluid.

H. notes that the highest incidence and mortality occurred in the month of January. During the two previous years the largest number of cases and deaths occurred in February. 64 o/o of the cases occurred during the first four months of the year.

The average age of the patients was 21.5 years. 64 o/o were below 20 years of age. In decades beyond the age of 25, the incidence progressively decreased.

Mortality was 36.2 o/o. (In the first year of the war it was 52.9 o/o and in the second, 35.6 o/o.)

Day of death : 1st day, 5 cases; 2nd day, 4 cases; 3rd day, 7 cases; 4th day, 8 cases; 5th day, 2 cases; 6th day, 2 cases; 2nd week, 5 cases; 3rd week, 10 cases; 4th week, 3 cases; 5th week, 2 cases; 6th week, 2 cases; and single cases on the 57th and 67th days. More than half of the cases were fatal during the first 6 days.

*Clinical Aspects.* — Initial symptoms were malaise, fever, headache, vomiting, and progressive increase of nervous symptoms. Within 24 hours the condition was highly suggestive. In some cases the onset was more gradual : in others it was startlingly acute—maniacal, convulsive, or accompanied with fainting and unconsciousness. In 2 cases the patients were found dead. In the second and third years of the war, 5 o/o of all cases (247) were characterized by the abruptly acute type of onset. Out of these 12 cases 6 proved fatal.

In cases with a gradual onset preceded by catarrh, it was impossible to say whether the catarrh was the commencement of the disease or whether the meningococcic invasion was superimposed over an influenza or catarrh. A considerable number of cases (31) occurred within three weeks after the change from civilian to military life. The author thinks this fact may point to a systemic invasion by the meningococcus.

Rashes due to the cerebro-spinal fever occurred in 60 o/o of the cases. The characteristic rash was hemorrhagic, especially in the acute or fulminating cases.

*Herpes labialis* occurred in 24 o/o of the cases, and was apparently a favorable sign.

Secondary infections occurred in 2 o/o of the cases.

For the purpose of the report only cases in which the meningococcus was demonstrated in the spinal fluid are considered. The author believes, however, that in doubtful cases serum should be administered pending the test. He notes that a clear fluid drawn by the first puncture is not a proof excluding cerebro-spinal meningitis. It does not follow, either, that a turbid fluid drawn after serum has been injected is positive proof of infective meningitis, because the serum may cause a polymorphonuclear exudation.

*Treatment.* — Out of 26 cases which did not receive serum treatment there were 14 deaths (53.6 o/o). Among the 117 cases treated by some form of serum, there was a mortality of 32.4 o/o. This result was practically the same as in the previous year, when among 95 cases treated by serum the mortality was 31.6 o/o. During the first year of the war, however, when an apparently inert serum was used, and when Flexner's serum was not available, the mortality for 105 cases treated by serum was 61 o/o.

Flexner's serum has appeared to give the best result. During the second and third years of the war 143 cases were treated by Flexner's serum either alone or in combination with other sera : among these the mortality was 28.6 o/o. Among the 109 cases treated by Flexner's serum alone, the mortality was 27.5 o/o. Among the 34 cases treated by Flexner's serum combined with some other, the mortality was 32.3 o/o.

During the third year of the war, Flexner's serum was used alone in 87 cases with a mortality of 29.2 o/o. Among 22 cases in which Flexner's serum was used in combination with others, mortality was 36.4 o/o. Among 13 cases treated by sera other than Flexner's the mortality was 46.3 o/o.

Among the 96 cases treated by serum within the first three days, the mortality was 32 o/o. Among 17 cases in which the treatment was commenced between the fourth and seventh days, it was 41 o/o. Among four cases treated after the seventh day there were no deaths. The author regards these observations as too few to contradict Flexner's conclusions, based upon 1211 cases, that mortality rises progressively with delay. He remarks that cases in which the treatment is likely to be given later than the seventh day are probably light ones which might recover spontaneously.

As to the number of intrathecal injections administered, it appeared that the best results were obtained in cases receiving from 2 to 6 injections. Among these there was a mortality of 26 o/o. Among those receiving 1 injection or more than 6, the mortality was 50 o/o or more. There was no evidence that the hypodermic or intramuscular injection enhances the effect of the intrathecal injection.

Serum rashes occurred in 60 o/o of the 96 cases which survived more than ten days. They usually appeared ten days after the first injection. Cases in which Flexner's serum was used were especially prone to the rash. The author thinks this fact may be due to the reaction caused in British subjects by a serum produced from horses in America. The quantity of serum administered seemed to have no influence upon the incidence of the rash. In 15.5 o/o out of 58 cases, the serum rash was preceded or accompanied by a recrudescence of meningeal symptoms which were relieved by lumbar puncture. This appearance can be distinguished from a genuine recurrence of meningococcic infection by tests of the fluid for the organism and for the normal reducing agent (glucose) which is absent in a genuine relapse. The mortality among cases with serum rash was 12 o/o with an average duration of 26 days.

Accidents due to lumbar puncture were rare. They were due to causes which should easily be avoided : increased intracranial pressure, anaphylaxis, and infection through the puncture.

